# Layer-dependent Schottky contact at TaX_2_–BY (X = S, Se, Te; Y = P, As, Sb) van der Waals interfaces

**DOI:** 10.1039/d4na00688g

**Published:** 2024-11-27

**Authors:** Israr Ul Haq, A. Mustaqeem, B. Ali, M. Umair Ashraf, U. Khan, Muhammad Idrees, M. Shafiq, Yousef Mohammed Alanazi, B. Amin

**Affiliations:** a Department of Physics, Abbottabad University of Science & Technology Abbottabad 22010 Pakistan binukhn@gmail.com +92-333-943-665; b Institute for Applied Physics, Department of Physics, University of Science and Technology Beijing Beijing 100083 China; c School of Physics and Electronic Engineering, Jiangsu University Zhenjiang 212013 Jiangsu China; d College of Engineering, Department of Chemical Engineering, King Saud University Riyadh Saudi Arabia

## Abstract

The mechanical, thermal and dynamical stabilities, electronic structure, contact type, and height of the barrier at the interface of TaX_2_ (X = S, Se, Te) and BY (Y = P, As, Sb) metal–semiconductor (MS) contact are investigated *via* first principles calculations. Binding energies, mechanical properties, phonon spectra and *ab initio* molecular dynamics (AIMD) simulations confirm the stabilities of these systems. TaX_2_–BY (X = S, Se, Te; Y = P, As, Sb) MS van der Waals heterostructures (vdWHs) are found to be metal with a Schottky contact at the interface. Formation of the n-type Schottky contact at the interface of TaX_2_–BY (X = S, Se, Te; Y = P, As, Sb) MS vdWHs favors electron conduction over hole conduction. Small (higher) effective mass (carrier mobility) make TaS_2_–BSb, TaSe_2_–BSb and TaTe_2_–BSb MS vdWHs, potential candidates for high speed nanoelectronic applications. Bader charge analysis shows that at the interface of TaX_2_–BY (X = S, Se, Te; Y = P, As, Sb) MS vdWHs, in TaX_2_ (BP, BAs) the electrons transfer from the TaX_2_ layer to the BP and BAs layer, while in TaX_2_ (BSb) the electrons transfer from the BSb layer to TaX_2_ layer.

## Abstract 2

The mechanical, thermal and dynamical stabilities, electronic structure, contact type, and height of the barrier at the interface of TaX_2_ (X = S, Se, Te) and BY (Y = P, As, Sb) metal–semiconductor (MS) contact are investigated .

## Graphical abstract

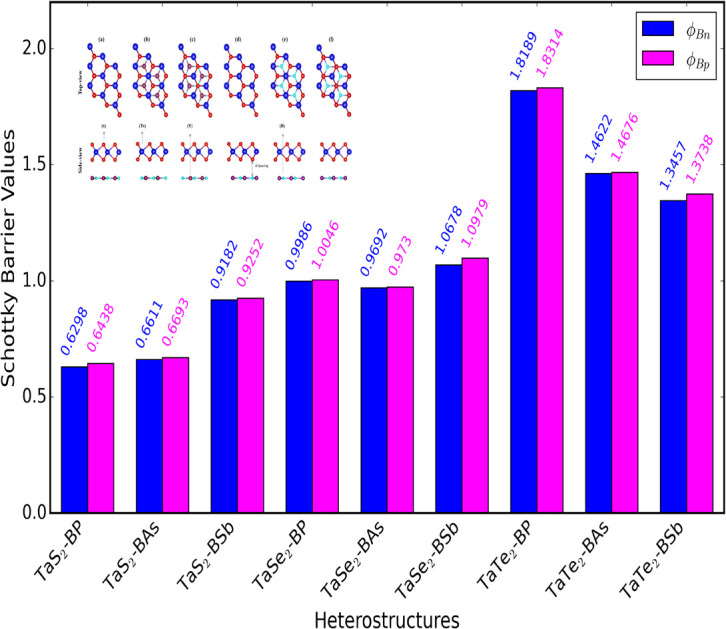

## 1. Introduction

After the successful development of graphene in 2004,^1^ researchers isolated and studied more than a dozen 2D materials within less than a decade.^2^ Therefore, the family of 2D materials extended from the carbon materials (graphene) to transition metal dichalcogenides (TMDCs),^3^ MXene,^4^ layered metal oxides^5^ and many more^6^ with insulating,^7^ semiconducting,^8^ semimetallic,^9^ metallic^10^ and superconducting nature.^11^ The significant interest in 2D materials is attributed to their special physical characteristics that emerge *via* confinement of the transport of heat and charge to a plane.^12^ The unique physical characteristics are expected to make a significant impact across various applications,^13^ spanning from high-performance sensors,^14^ storage,^15^ catalysis,^16^ inert coating,^17^ electronic,^18^ optoelectronic^19^ and spintronic^20^ devices. In the family of 2D materials, TMDCs with MX_2_ (M = transition metal atom, X = chalcogen atom) general formula are of particular importance, where weak van der Waals (vdW) forces hold these layers together, allowing for their easy extraction from the bulk.^21^ The simplicity of their preparation and diverse range of characteristics, make them noteworthy in the realm of 2D materials.^22^ Similarly, another class of 2D materials based on group III–V semiconductors has been explored both theoretically^23,24^ and experimentally.^25,26^ The direct band gap and hexagonal lattice structure, identical to graphene, render these materials promising for the next generation of nano^27^ and optoelectronic device^28^ applications.

The physical properties of the aforementioned 2D materials come with several key challenges^29^ such as scalability, the production of high-quality materials with large surface area, and high cost. Current synthesis methods, like chemical vapor deposition (CVD), can be expensive and complex. Stability and durability of some 2D materials can be chemically reactive or sensitive to environmental factors such as air and moisture, which can degrade their properties over time.^30,31^ Therefore, controlling the electronic properties and band gap of 2D materials is crucial, while some 2D materials are natural semiconductors, others, like graphene, require engineering to open a band gap for specific applications. Consequently, researchers have focused on the tuning of these materials *via* several techniques,^32,33^ for their useful device applications.^19^ In these techniques, stacking of 2D materials in the form of van der Waals heterostructures (vdWHs)^34^ provides a flexible platform for exploring novel phenomena in the design of nanoelectronic devices.^35^ Numerous vdWHs, particularly those in the form of semiconductor–semiconductor (SS) contact, have been extensively studied both theoretically^36,37^ and experimentally^38,39^ for their promising and remarkable applications in the field of optoelectronic devices.^40^ The Schottky barrier (SB) in the metal and semiconductor (MS) vdWHs minimizes resistance at contact, hence tuning (enhancing) the polarity (selectivity) of carriers in the transistor channel (photovoltaic cells), therefore, playing a crucial role in devices.^41,42^ These contacts (SS and MS) are fabricated *via* ultra-thin and flat surfaces without defects and with outstanding chemical (mechanical) stability (flexibility).^43^

Although, TMDCs have already been used in almost every MS contact,^44–50^ a significant gap exists regarding the exploration of 2D TaX_2_ (X = S, Se, Te) and BY (Y = P, As, Sb) MS contact. Therefore, in the present work, we have focused on the modelling of TaX_2_–BY MS vdWHs. Structural stability, electronic structure, contact type and height of the barrier of the modelled heterostructures are investigated, with the goal of exploring the potential applications of these materials in device fabrications.

## 2. Computational details

We used density functional theory (DFT) with the PWSCF code^51^ with generalized gradient approximation (GGA) in the Perdew–Burke–Ernzerhof (PBE) style.^52^ We fixed the convergence criteria for force to 10^−3^ Å^−1^ and energy to 10^−5^ eV for the optimization/relaxation of the lattice constant/atomic positions. We also fixed the cut-off energy to 800 eV and used a 16 × 16 × 1 *k*-grid for the Brillouin zone integration. To mitigate interactions between neighbouring layers of atoms, we set a vacuum layer with a thickness of 25 Å.

Mechanical stability of these systems are examined using the energy-strain method in the VASPKIT code^53^ and visualized with the ELATE software.^54^ Thermal (dynamical) stabilities were investigated *via* AIMD (phonon) simulation (calculations).^55,56^ In the case of thermal stability, *ab initio* molecular dynamics (AIMD) simulations,^55^ through the Nose thermostat algorithm at a temperature of 300 K for a total of 6 ps with a time interval of 1 fs are performed. For dynamical stability, we used a 3 × 3 × 1 supercell consisting of 45 atoms. We have also used density functional perturbation theory (DFPT) with the VASP code^57^ to determine the harmonic second-order interatomic force constants (IFCs). In addition, we employed the PHONOPY code^58^ to calculate the phonon dispersion using the frozen phonon approximation.

Type (height) of the Schottky contact (barrier) is obtained using first principles calculations^59^ using *Φ*_Bn_ = *E*_CBM_ − *E*_F_ and *Φ*_Bp_ = *E*_F_ − *E*_VBM_, where *E*_CBM_ and *E*_VBM_ are the energies of the band edges of the semiconducting material and *E*_F_ is the Fermi level of the metallic material. Type (n and p) of the Schottky barrier without Δ*V* were also calculated^60^ using the Schottky–Mott rule; *ϕ*_Bn_ = *ϕ*_(metallic-monolayer)_ − *χ*_(MS-vdWH)_ and *ϕ*_Bp_ = *I*_(MS-vdWH)_ − *ϕ*_(metallic-monolayer)_, here *ϕ*, *χ*, and *I* are the work function, electron affinity, and ionization energy, respectively, of the MS vdWHs and corresponding monolayers. Band bending was calculated^61^ using Δ*ϕ* = *ϕ*_(metallic-monolayer)_ − *ϕ*_(semiconducting-monolayer)_, where *ϕ*_(metallic-monolayer)_ and *ϕ*_(semiconducting-monolayer)_ denote the work function of the corresponding metallic and semiconducting monolayers, respectively, in the MS vdWHs.

## 3. Results and discussion

**Fig. 1:**
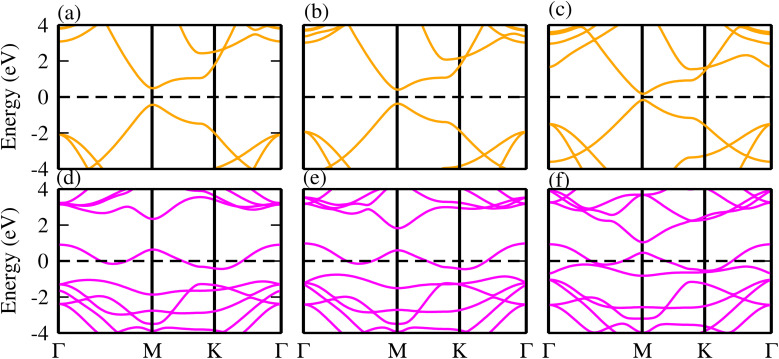
Electronic band structures of (a) BP, (b) BAs, (c) BSb, (d) TaS_2_, (e) TaSe_2_ and (f) TaTe_2_ monolayers.

Although, monolayers of TaX_2_ (X = S, Se, Te) and BY (Y = P, As, Sb) have already been investigated in detail in ref. 62 and 63. To verify our present approach, we have further calculated the electronic band structure of TaX_2_ (X = S, Se, Te) and BY (Y = P, As, Sb). In agreement with ref. 62 and 63, TaX_2_(BY) monolayers exhibit a metallic (direct bandgap semiconducting) nature (see Fig. 1).

**Fig. 2:**
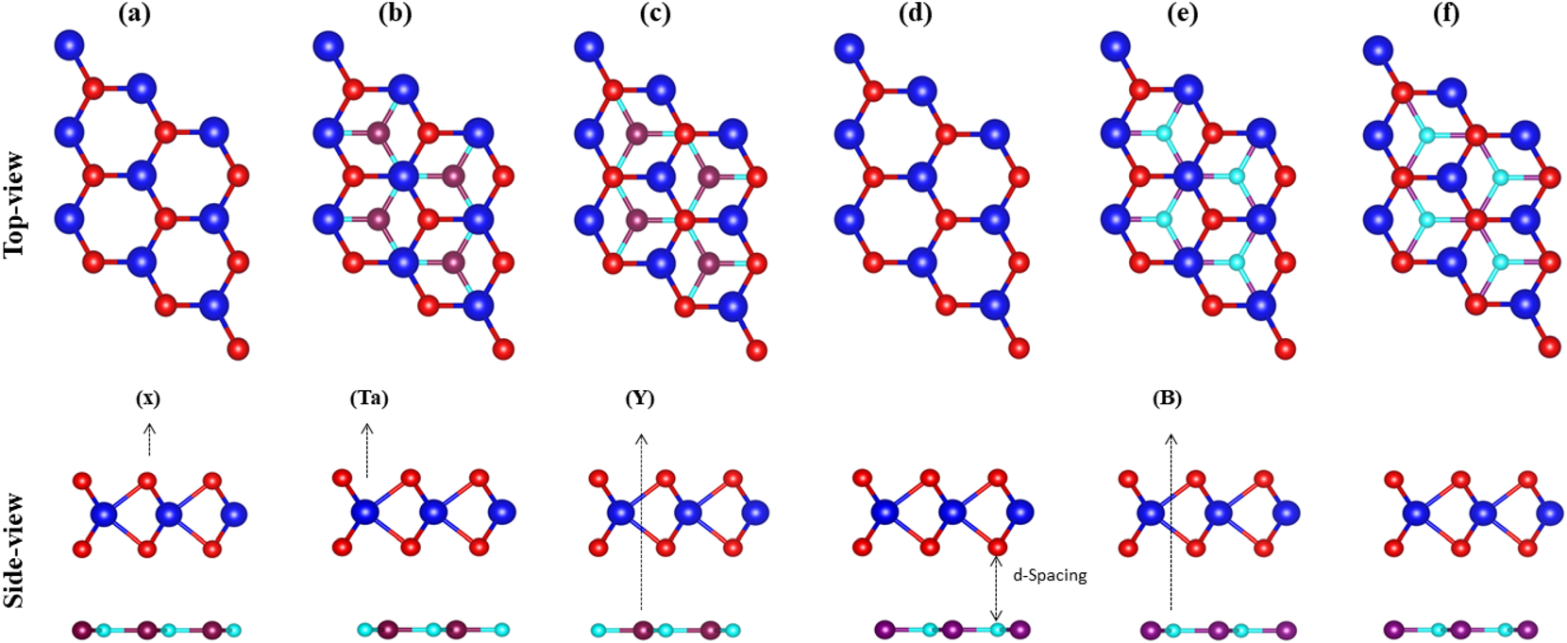
Possible stacking configurations of TaX_2_–BY (X = S, Se, Te; Y = P, As, Sb) MS vdWHs.

Therefore, using the optimized lattice constant of TaX_2_ (X = S, Se, Te)^64,65^ and BY (Y = P, As, Sb),^66,67^ TaX_2_–BY MS contact in six different vdWH stacking configuration patterns are fabricated, see Fig. 2. In (a), the Ta(X) atom of the TaX_2_ layer is placed on top of the B(Y) atom of the BY layer. In (b), the Ta atom of the TaX_2_ layer is placed on top of the B atom of the BY layer, while the X(Y) atom of the TaX_2_(BN) layer is placed on the hexagonal centre. In (c), the X atom of the TaX_2_ layer is placed on top of the B atom of the BY layer, while the Ta(Y) atoms of TaX_2_(BY) are placed on the hexagonal centre. In (d), the Ta atom of the TaX_2_ layer is settled on top of the Y atom of the BY layer. In (e), the Ta atom of the TaX_2_ layer is stacked on top of the Y atom of the BY layer, while the X(B) atom of TaX_2_(BY) is placed on the hexagonal centre. In (f), the X atom of the TaX_2_ is settled on top of the Y atom of BY, while Ta(B) atoms are placed on the hexagonal centre.

Binding energy^68^ and the interlayer distance (d) of all six patterns of TaX_2_–BY MS vdWHs are calculated and presented in Table 1. Stacking (b) of TaS_2_–BP, (b) of TaS_2_–BAs, (b) of TaS_2_–BSb, (e) of TaSe_2_–BP, (b) of TaSe_2_–BAs, (b) of TaSe_2_–BSb, (e) of TaTe_2_–BP, (e) of TaTe_2_–BAs and (d) of TaTe_2_–BSb, are found to be the most stable stacking configurations, based on the most smaller (higher) interlayer distance (binding energies),^68^ see Table 1. The variation in stable stacking is due to the induced strain from the distinct chalcogen atoms and the dissimilar interface atoms. The optimized lattice constant and bond length of the most stable stacking configurations are presented in Table 2. Before considering the stable stacking configurations for further investigation, we further confirmed the stability *via* calculating the mechanical properties of these systems.

**Table 1:** Binding energy (*E*_b_ in eV) and interlayer distance (*d* in Å) of TaX_2_–BY (X = S, Se, Te; Y = P, As, Sb) MS vdWHs in (a–f) stacking configurations

TaX_2_–BY		TaS_2_–BP	TaS_2_–BAs	TaS_2_–BSb	TaSe_2_–BP	TaSe_2_–BAs	TaSe_2_–BSb	TaTe_2_–BP	TaTe_2_–BAs	TaTe_2_–BSb
(a)	*E*_b_	−0.010	−0.013	−0.025	−0.011	−0.013	−0.019	−0.011	−0.014	−0.019
	*d*	3.549	3.542	3.062	3.620	3.638	3.574	3.889	3.834	3.811
(b)	*E*_b_	−0.016	−0.019	−0.031	−0.017	−0.021	−0.028	−0.018	−0.023	−0.030
	*d*	3.329	3.327	3.084	3.711	3.406	3.558	3.874	3.880	3.913
(c)	*E*_b_	−0.011	−0.014	−0.024	−0.012	−0.015	−0.020	−0.012	−0.015	−0.020
	d	3.378	3.385	3.657	3.444	3.454	3.642	3.626	3.601	3.766
(d)	*E*_b_	−0.012	−0.016	−0.025	−0.014	−0.016	−0.021	−0.015	−0.018	−0.049
	*d*	3.339	3.650	3.639	3.392	3.740	3.606	3.536	3.493	2.293
(e)	*E*_b_	−0.015	−0.019	−0.030	−0.018	−0.021	−0.027	−0.019	−0.023	−0.029
	*d*	3.638	3.661	3.628	3.317	3.765	3.781	3.476	3.486	3.950
(f)	*E*_b_	−0.010	−0.013	−0.024	−0.010	−0.013	−0.018	−0.010	−0.012	−0.017
	*d*	3.576	3.585	3.612	3.738	3.694	3.599	3.934	3.938	3.894

**Table 2:** Lattice constant (*a* in Å), bond length (in Å), interlayer distance (*d* in Å), elastic constants (*C*_11_ and *C*_12_ in N m^−1^), Young's modulus (*E* in N m^−1^), shear modulus (*G* in N m^−1^), Poisson's ratio (*ν*), work function (*ϕ* in eV), potential (Δ*V* in eV), effective mass of the carriers 
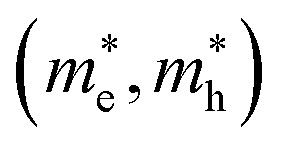
, ionization energy (*I* in eV), electron affinity (*χ* in eV), and band bending (Δ*W* in eV) of TaX_2_–BY (X = S, Se, Te; Y = P, As, Sb) MS vdWHs

TaX_2_–BY	TaS_2_–BP	TaS_2_–BAs	TaS_2_–BSb	TaSe_2_–BP	TaSe_2_–BAs	TaSe_2_–BSb	TaTe_2_–BP	TaTe_2_–BAs	TaTe_2_–BSb
*a*	3.26	3.35	3.51	3.34	3.43	3.61	3.46	3.54	3.72
M–X	2.46	2.48	2.52	2.58	2.60	2.64	2.79	2.79	2.78
B–N	1.88	1.94	2.08	1.93	1.98	2.10	1.99	2.05	2.32
*d*	3.33	3.33	3.08	3.32	3.41	3.56	3.48	3.49	2.29
*C*_11_	299.38	282.34	252.80	254.77	252.49	226.39	142.89	156.02	159.40
*C*_12_	84.46	82.65	73.65	79.53	69.60	64.49	62.46	72.88	42.81
*E*	275.55	258.15	231.34	299.95	233.31	208.02	115.58	121.98	147.90
*G*	107.46	99.85	89.57	87.62	91.45	80.95	40.21	41.57	58.29
*ν*	0.282	0.239	0.291	0.312	0.276	0.285	0.437	0.467	0.269
Δ*V*	4.93	2.22	4.88	7.23	4.41	2.67	4.74	1.64	2.53
*ϕ*	4.01	3.92	3.65	3.81	3.82	3.63	3.28	3.58	4.01
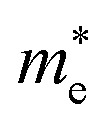	0.71	0.61	0.43	0.79	0.68	0.48	0.94	0.79	0.57
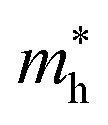	0.66	0.57	0.41	0.75	0.65	0.47	0.91	0.77	0.55
*I*	4.65	4.59	4.58	4.82	4.79	4.73	5.11	5.05	5.38
*χ*	4.65	4.58	4.57	4.81	4.79	4.70	5.10	5.04	5.36
Δ*W*	0.23	0.23	0.23	0.24	0.24	0.24	0.32	0.29	0.24

Mechanical stability of the most stable stacking configurations of TaX_2_–BY (X = S, Se, Te; Y = P, As, Sb) MS vdWH are assisted *via* the energy-strain method.^69–71^ Using the hexagonal symmetry of TaX_2_–BY MS vdWH, two independent elastic constants (*C*_11_ and *C*_12_), Young's modulus, shear modulus, and Poisson's ratios are calculated and presented in Table 2, that meet the Born criteria: *C*_11_ > 0, *C*_12_ > 0, and *C*_11_ > ∣*C*_12_∣.^69–71^ Therefore, these findings indicate the high mechanical stability of TaX_2_–BY (X = S, Se, Te; Y = P, As, Sb) MS vdWH, in agreement with previous reports.^69–71^

**Fig. 3:**
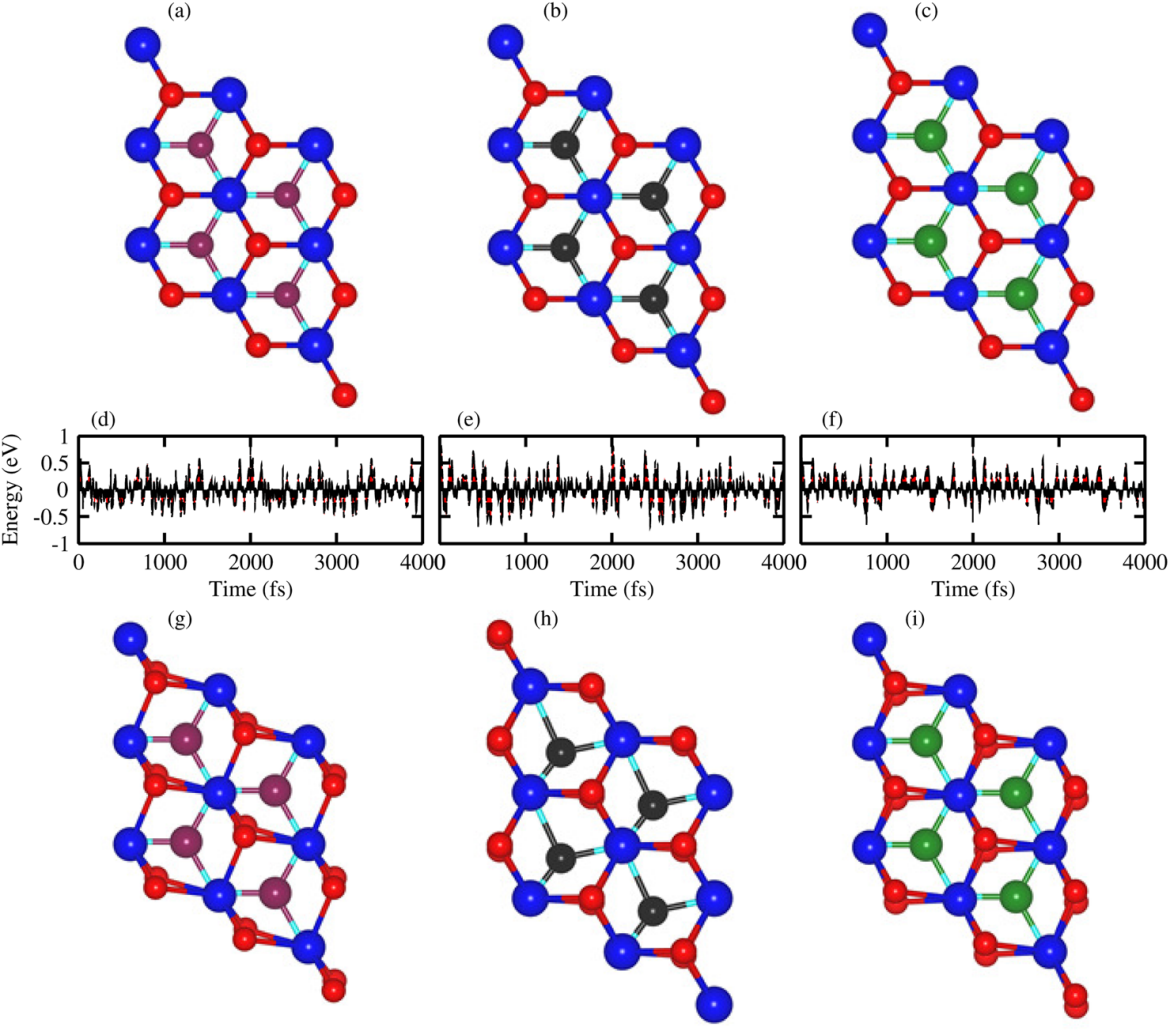
*Ab initio* molecular dynamics simulation of (a, d, and g) TaS_2_–BP, (b, e, and h) TaS_2_–BAs and (c, f, and i) TaS_2_–BSb, van der Waals heterostructures. Geometrical structure (a–c) before heating; (g–i) after heating with (d–f) fluctuating energy.

Thermal stability in terms of the fluctuation energy as a function of time, of the energetically most favorable stacking configurations in TaX_2_–BY (TaS_2_–BP, TaS_2_–BAs and TaS_2_–BSb), are given in Fig. 3. One can observe that in the case of TaS_2_–BP, TaS_2_–BAs and TaS_2_–BSb, heterostructures after 4 ps, there is no structure distortion. Moreover, through the AIMD simulation, our results demonstrate that the geometrical structure of TaS_2_–BP, TaS_2_–BAs and TaS_2_–BSb is retained after 4000 step simulations and the average value of the total energy remains nearly constant. All these findings demonstrate that TaS_2_–BP, TaS_2_–BAs and TaS_2_–BSb are thermally stable at room temperature.

**Fig. 4:**
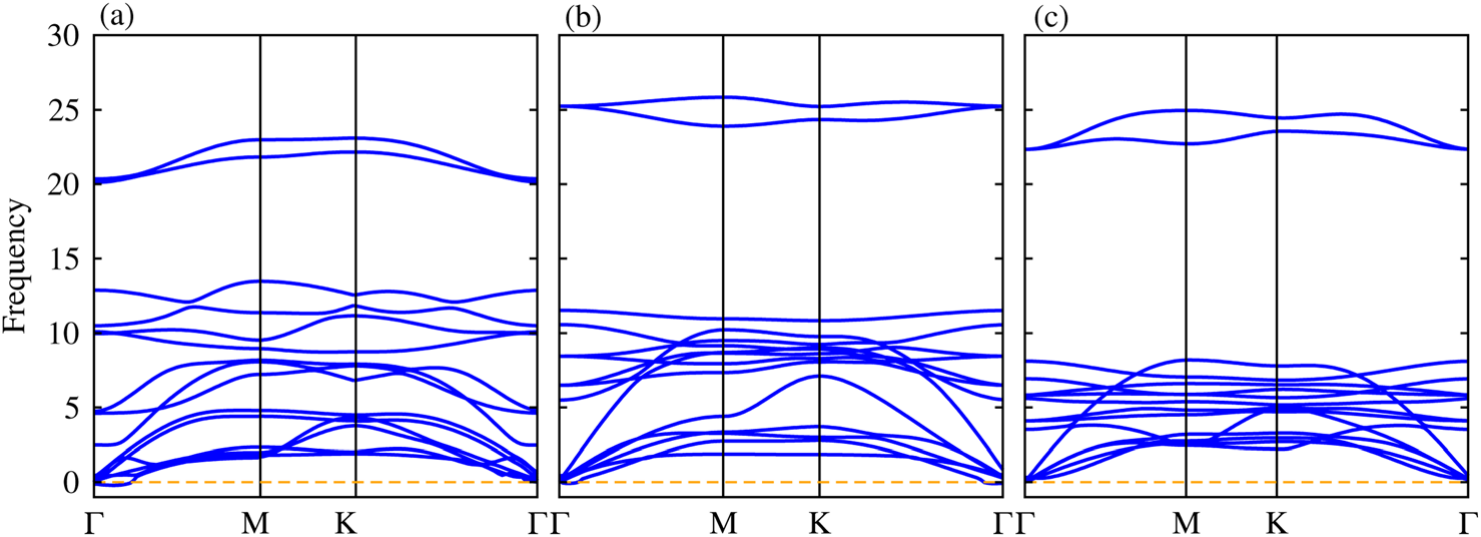
Phonon dispersion curves of (a) TaS_2_–BP, (b) TaS_2_–BAs and (c) TaS_2_–BSb MS vdWHs.

**Fig. 5:**
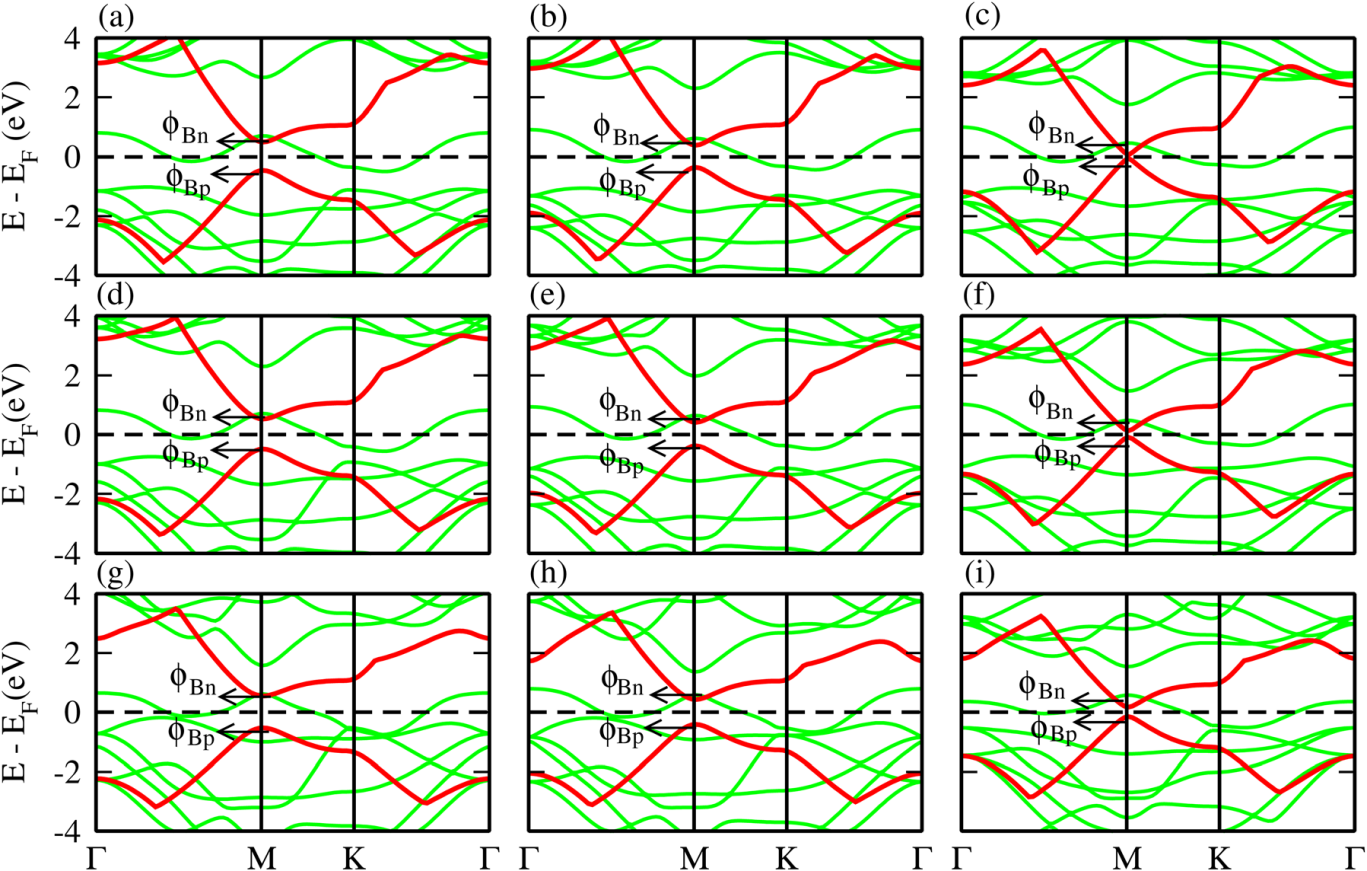
Electronic band structure of (a) TaS_2_–BP, (b) TaS_2_–BAs, (c) TaS_2_–BSb, (d) TaSe_2_–BP, (e) TaSe_2_–BAs, (f) TaSe_2_–BSb, (g) TaTe_2_–BP, (h) TaTe_2_–BAs and (i) TaTe_2_–BSb MS vdWHs.

The phonon dispersion curves in Fig. 4 for the TaS_2_–BP, TaS_2_–BAs, and TaS_2_–BSb MS vdWHs show no imaginary frequencies throughout the Brillouin zone, confirming their dynamic stability. The lack of imaginary modes indicates that the atomic arrangements reside at a local minimum on the potential energy surface, which is a key indicator of structural stability. Using the PBE functional, the electronic band structures are displayed in Fig. 5, where green (red) lines are attributed to the TaX_2_(BY) layers in TaX_2_–BY (X = S, Se, Te; Y = P, As, Sb) MS vdWHs. The electronic band structures in Fig. 5, show that all TaX_2_–BY (X = S, Se, Te; Y = P, As, Sb) MS vdWHs are metals with type-III band alignment^72^ and look like the sum of band structure of TaX_2_ and BY (Y = P, As, Sb) monolayers. The transition of BY (Y = P, As, Sb) to a metal is ascribed to the contribution of metallic states from TaX_2_ (X = S, Se, Te) monolayer, enhancing its conductivity and making it suitable for various electronic applications involving Schottky contacts.

**Fig. 6:**
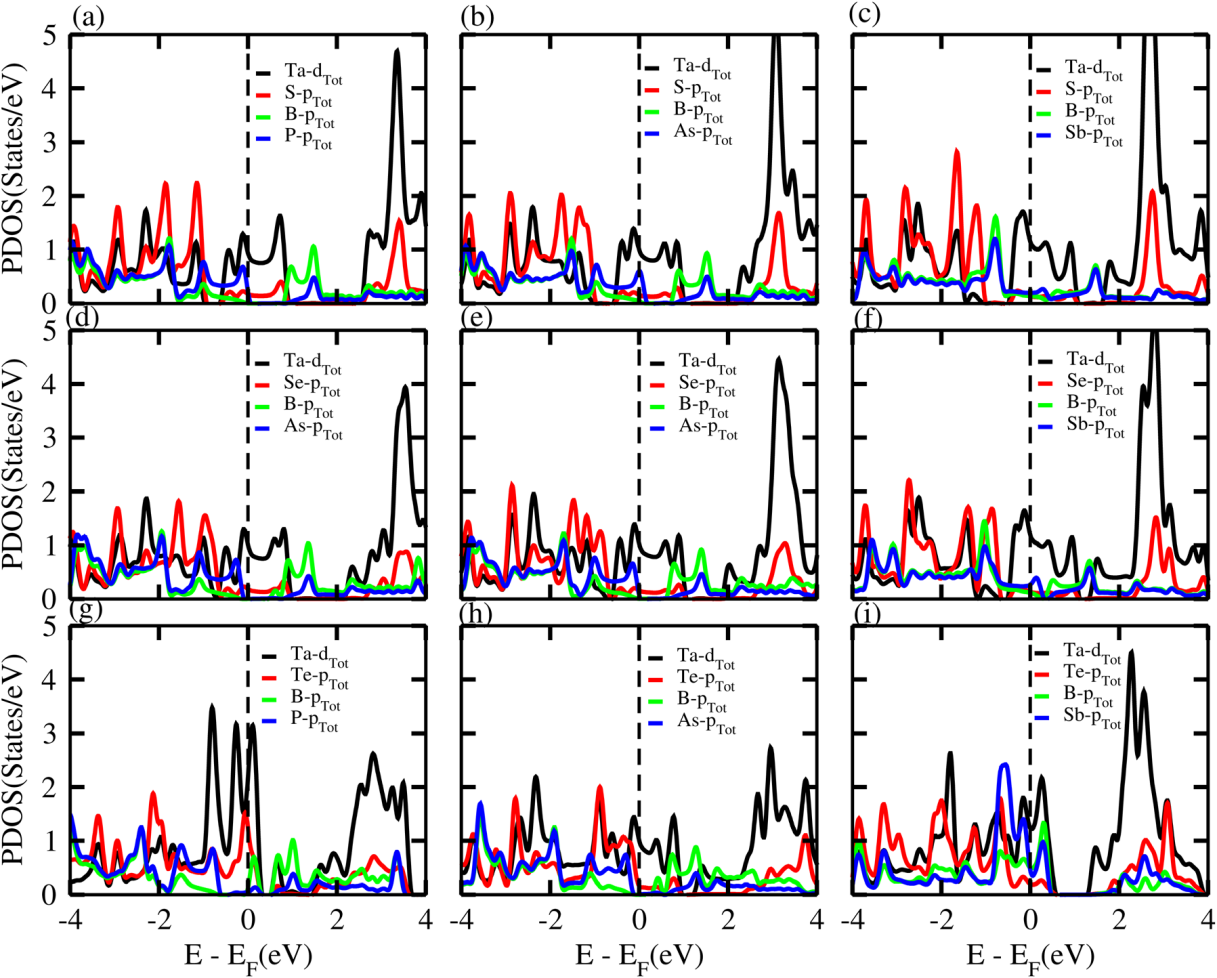
PDOS of (a) TaS_2_–BP, (b) TaS_2_–BAs, (c) TaS_2_–BSb, (d) TaSe_2_–BP, (e) TaSe_2_–BAs, (f) TaSe_2_–BSb, (g) TaTe_2_–BP, (h) TaTe_2_–BAs and (i) TaTe_2_–BSb, vdWHs.

PDOS of the TaX_2_–BY MS vdWHs are presented in Fig. 6, and show that the main contributions at the Fermi level are due to the Ta-d (d_3*z*^2^−*r*^2^_, d_*xy*_) and (S, Se, Te)-p orbital of the TaX_2_ monolayers (crossing the Fermi level); while both the valence and conduction band without crossing the Fermi level, are due to the B-p_*x*_ and (Y = P, As, Sb)-p_*z*_ orbitals of the BY monolayer. After stacking, the TaX_2_ monolayer shifts the conduction band minimum (CBM) of the BY layer toward the Fermi level due to the difference in electronegativity. The position of these orbitals near to the Fermi level in the valence and conduction bands show that the band structure of BY and TaX_2_ monolayers are well preserved in the TaX_2_–BY MS vdWHs. The minimum (maximum) of the conduction (valence) band is due to the Ta-d_3*z*^2^*r*^2^_ (Ta-d_*x*^2^−*y*^2^_, d_*xy*_) orbital with a very small contribution from the Ta-d_*x*^2^−*y*^2^_ and d_*xy*_ orbitals, see Fig. 6. The B-p_*x*_ and p_*y*_ orbital dominate the valence band minimum (VBM), with a small contribution from the B-p_*z*_ orbitals. Through modelling TaX_2_–BY MS vdWHs, we found that strain is induced in the corresponding monolayers due to the lattice mismatch, tuning the coupling between the orbitals of the TMDCs (Ta-d) and chalcogen atoms ((S, Se, Te)-p). Therefore, splitting in the bonding and antibonding states of BY monolayers in TaX_2_–BY MS vdWHs fluctuates, and hence the position of the contributing orbital at the Fermi level varies.

Furthermore, for the use of TaX_2_–BY (X = S, Se, Te; Y = P, As, Sb) MS vdWHs in device applications, effective mass and carrier mobility are investigated and related by μ = *eτ*/m*.^73^ Thus, to evaluate the carrier mobility of TaX_2_–BY MS vdWHs, effective mass is calculated using^74^
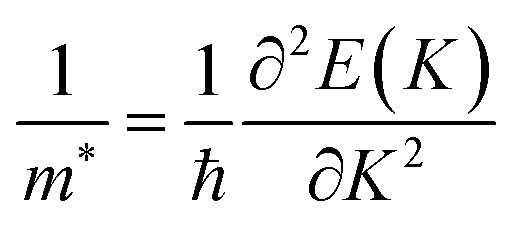
 where, *ℏ* is Planck’s constant and *K* is the wave vector. Based on the above equation, using the parabolic fitting of the CB and VB of TaX_2_–BY MS vdWHs, the calculated effective mass for electrons (holes) are given in Table 2. Quite a small effective mass in the case of TaS_2_–BSb, TaSe_2_–BSb and TaTe_2_–BSb MS vdWHs, exhibit high carrier mobility, and hence show potential for efficient nanoelectronic devices.

**Fig. 7:**
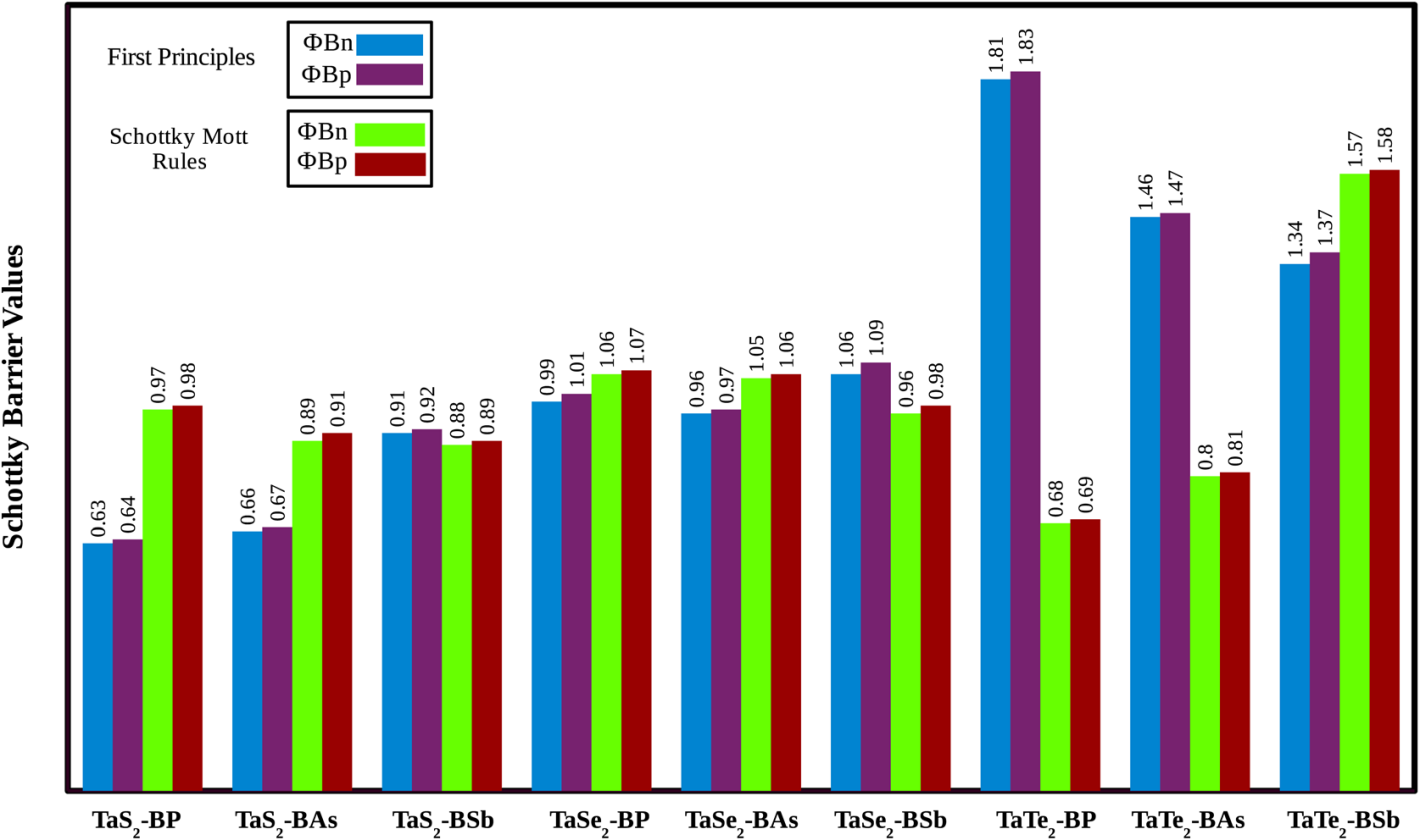
Schottky barrier height calculated using the Schottky–Mott rule and first principles calculation of TaX_2_ (X = S, Se, Te)–BY (Y = P, As, Sb) MS vdWHs.

Relative locations of the metal (TaX_2_) with semiconductor (BY) band edges in TaX_2_–BY (X = S, Se, Te; Y = P, As, Sb) MS vdWHs establish the Schottky or ohmic junctions across the interface. Tuning the Schottky barrier (SBH) can change the current flow across the interface of the TaX_2_–BY (X = S, Se, Te; Y = P, As, Sb) MS vdWHs, hence boosting the device performance.^75,76^ The band structures in Fig. 5 indicate that the Fermi level of TaX_2_ (metal layer) sits in the band edges of BY (semiconductor layer), and hence establish Schottky contacts^77^ at the interface of TaX_2_–BY MS vdWHs. The type of Schottky contact (n-type or p-type) is obtained using the Schottky–Mott rule,^78^ see computational details. The calculated values of the Schottky barrier *ϕ*_Bn_ and *ϕ*_Bp_ using first principles calculations and the Schottky–Mott rule, are given in Fig. 7, which shows that *ϕ*_Bn_ of all stacking configurations is smaller than *ϕ*_Bp_, suggesting that they form an n-type Schottky contact. Therefore, the MS vdWHs under consideration favor electron conduction over hole conduction, and hence have significant implications in transistors, sensors, photodetectors, and other electronic components.^79^ It is clearly observed that the SBH increases (decreases) from P to As to Sb in TaS_2_–BY (TaTe_2_–BY) MS vdWHs. Moreover, the metallic nature of these heterostructures suppress the metal induced gap states (MIGS) in BY (Y = P, As, Sb) monolayers, hence, leading to weak Fermi Level Pinning (FLP). Weak vdW interactions redistribute the charge density at the interface, see Fig. 9 (discussed later on), and hence establish interface dipoles (ID). Therefore, neglecting the metal semiconductor interaction (without considering Δ*V*) should ideally follow the predictions of the Schottky–Mott rule. Therefore, type (n and p) of the Schottky barrier without Δ*V* were also calculated^60^ using *ϕ*_Bn_ = *ϕ*_(metallic-monolayers)_ − *χ*_(vdWH)_ and *ϕ*_Bp_ = *I*_(vdWH)_ − *ϕ*_(metallic-monolayers)_. These IDs may lead to deviation from the Schottky–Mott limit, due to shifting of the electronic levels and FLP effect at the interface from their original positions.^80^ The band bending of the TaX_2_–BY systems reveals the presence of both p-type and n-type Schottky barrier even without the transfer of charge between the corresponding monolayers. When Δ*ϕ* are greater than 0 or Δ*ϕ* is less than 0, it signifies the formation of n-type and p-type Schottky contacts.^50^ In the TaX_2_–BY vdWHs under study, Δ*ϕ* is greater than 0, see Table 2. This observation suggests that charge will preferentially flow from metal to semiconductor, indicating n-type contact due to the high values of Δ*ϕ* (greater than 0), which demonstrate that holes will flow from TaX_2_ to the BY layer. The controllable electronic properties and formation of the Schottky contact in TaX_2_–BY MS vdWHs makes it a potential candidate for Schottky nanodevices.^50^

**Fig. 8:**
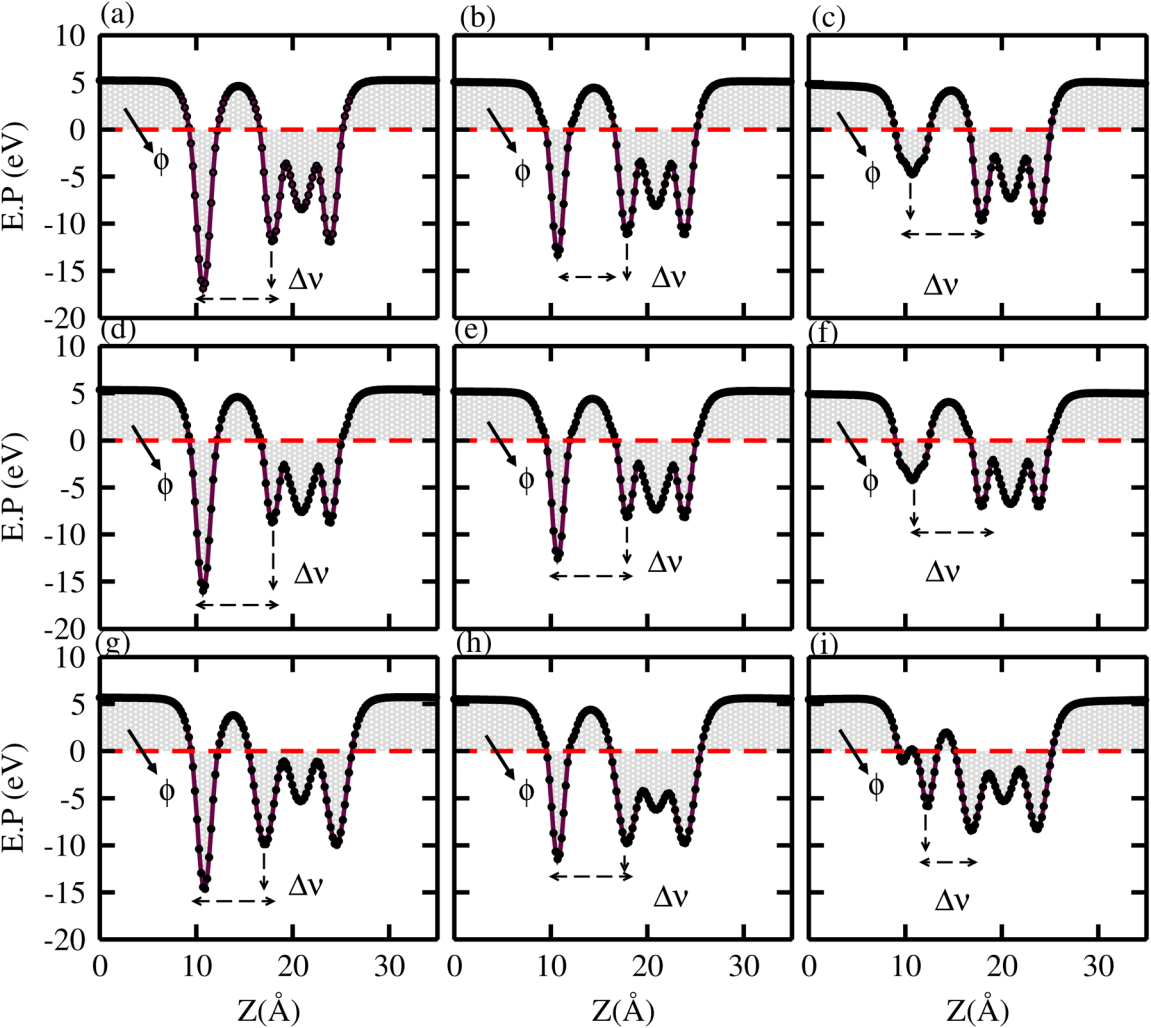
Average electrostatic potential of (a) TaS_2_–BP, (b) TaS_2_–BAs, (c) TaS_2_–BSb, (d) TaSe_2_–BP, (e) TaSe_2_–BAs, (f) TaSe_2_–BSb, (g) TaTe_2_–BP, (h) TaTe_2_–BAs, and (i) TaTe_2_–BSb, MS vdWHs.

In the case of TaX_2_–BY MS vdWHs, the TaX_2_ (BP, BAs) layer exhibits shallower (deeper) potential confirming charge transfer from TaX_2_ to the BP and BAs layer. On the contrary, in the case of TaX_2_ (X = S, Se, Te)–BSb, the TaX_2_ (BSb) layer is deeper (shallower), which confirms the transfer of charge from the BSb layer to TaX_2_, due to the fact that BSb has a higher work function than TaX_2_. The calculated Δ*V* varies within (1.64 to 7.23 eV) due to the difference in electronegativity among the atoms^81^ at the interface. For both TaX_2_ and BY monolayers in the form of TaX_2_–BY MS vdWHs, the electrostatic potential effectively controls charge movement at the interface, contributing to increased energy conversion efficiency. The work function (*ϕ*) of TaX_2_–BY MS vdWHs – defined as the difference between the vacuum level and Fermi level – was calculated using average electrostatic potential as displayed in Fig. 8. *ϕ* predominantly depends on the condition of the material surface due to changes in surface electric field settings and electron distribution at the interface. The calculated *ϕ* for of TaX_2_–BY MS vdWHs lies in the range 3.285–4.009 eV, see Table 2, which shows the potential for field effect transistors.^82^

**Fig. 9:**
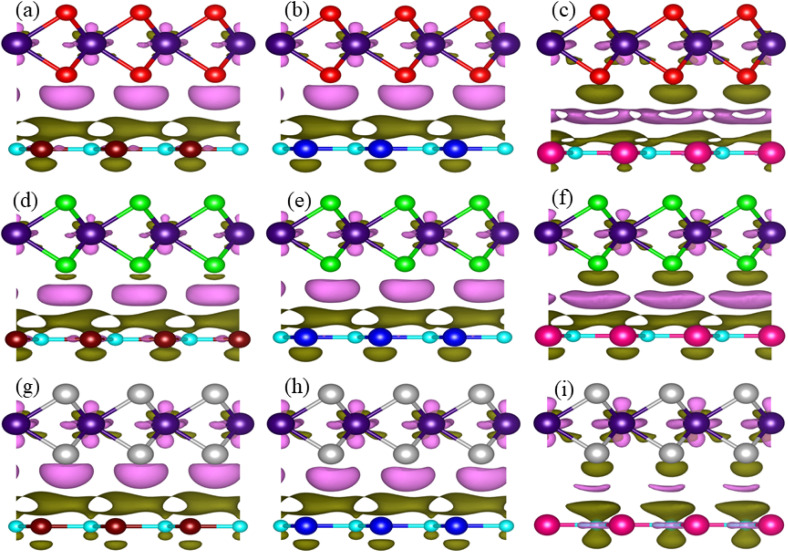
Charge density difference of (a) TaS_2_–BP, (b) TaS_2_–BAs, (c) TaS_2_–BSb, (d) TaSe_2_–BP, (e) TaSe_2_–BAs, (f) TaSe_2_–BSb, (g) TaTe_2_–BP, (h) TaTe_2_–BAs, and (i) TaTe_2_–BSb, MS vdWHs.

To see the contact charge redistribution, we have calculated the charge density difference of the TaX_2_–BY (X = S, Se, Te; Y = P, As, Sb) MS vdWHs using^83,84^ ▽*ρ* = *ρ*_MSvdWHs_ − *ρ*_monolayer-I_ − *ρ*_monolayer-II_. In the case of TaX_2_–BY (X = S, Se, Te; Y = P, As) MS vdWHs, charge depletion (accumulation) around the TaX_2_ (BP) layer indicates loss (gain) of electrons in TaX_2_ (BP, BAs). For TaX_2_–BSb MS vdWHs, TaX_2_ (BSb) gain (loss) of electrons in this region makes an electron (hole)-rich region.^85^ The BSb layer becomes a depletion region with fewer electrons, resulting in a hole-rich environment, while the TaX_2_ layer accumulates electrons and becomes electron-rich as shown in Fig. 9. Quantitative behaviour of charge transfer, analysed *via* Bader charges, show a maximum of 0.065409*e* and minimum of 0.000399*e* transferred from TaX_2_ to the BY monolayer, see Table 3. The phenomena of the transfer of charge shows a strong interlayer coupling and vdWh interaction established at the interface of TaX_2_ and BY monolayers in TaX_2_–BN MS vdWHs. A charge transportation built-in-electric field in the interface, creates a region where charges are separated, generating an electric field at the interface^86^ which enhances the carrier mobility along with the number of carriers (holes and electrons). A similar result is also confirmed *via* experiments in graphene/GaSe ^87^ and graphene/MoS_2_.^88^ Ionization potential (*I*); *I* = *E*_vac_ − *E*_VBM_, for the TaX_2_–BY MS contact in Table 2, helps in determining the Schottky barrier height, crucial for understanding the behavior of MS contacts.

**Table 3:** Charge redistribution at the interface of TaX_2_–BY (X = S, Se, Te; Y = P, As, Sb) MS vdWHs: e^−^ (h^+^) shows transfer of electrons (holes) from one layer to another

TaX_2_–BY	TaS_2_–BP	TaS_2_–BAs	TaS_2_–BSb	TaSe_2_–BP	TaSe_2_–BAs	TaSe_2_–BSb	TaTe_2_–BP	TaTe_2_–BAs	TaTe_2_–BSb
TaX_2_	0.026 (h^+^)	0.038 (h^+^)	0.065 (e^−^)	0.013 (h^+^)	0.024 (h^+^)	0.050 (e^−^)	0.001 (h^+^)	0.008 (h^+^)	0.033 (e^−^)
BY	0.027 (e^−^)	0.039 (e^−^)	0.065 (h^+^)	0.012 (e^−^)	0.025 (e^−^)	0.051 (h^+^)	0.001 (e^−^)	0.009 (e^−^)	0.032 (h^+^)

## 4. Conclusion

Using DFT calculations and electronic band structure, the contact (barrier) type (height) at the interface of TaX_2_–BY (X = S, Se, Te; Y = P, As, Sb) MS vdWHs are investigated. The stability of these systems are confirmed *via* binding energies, mechanical (born criteria), properties, AIMD simulation and phonon spectra calculations. Electronic band structures confirm the metallic behaviour of TaX_2_–BY MS vdWHs with weak vdW interactions in the corresponding monolayers. The quite small effective mass in the case of TaS_2_–BSb, TaSe_2_–BSb and TaTe_2_–BSb MS vdWHs, exhibit high carrier mobility and hence show potential for high speed nanoelectronic applications. n-type Schottky contact at the interface of TaX_2_–BY MS vdWHs favor electron conduction over hole conduction, hence has significant applications in transistors, sensors, photodetectors, and other electronic components. Large potential drop in the case of TaS_2_–BP, TaS_2_–BSb, TaSe_2_–BP, TaSe_2_–BAs and TaTe_2_–BP of the TaX_2_–BY MS vdWHs, suggests a considerable electrostatic field at the interface, which controls the charge transportation and strengthens the power conversion efficiency. Analysis of the Bader charges, show that a maximum (minimum) of 0.065409 (0.000399)*e* are transferred from the TaX_2_(BY) monolayer. The work function for the TaX_2_–BY MS contact lies in the 3.285–4.009 eV range, which shows potentiality for FET.

## Data availability

The data that support the findings of this study are available on request from the corresponding author.

## Conflicts of interest

There are no conflicts to declare.

## References

[cit1] Lee H. C., Liu W. W., Chai S. P., Mohamed A. R., Lai C. W., Khe C. S., Hidayah N. M. S. (2016). Procedia Chem..

[cit2] Geng D., Yang H. Y. (2018). Adv. Mater..

[cit3] Li H., Shi Y., Chiu M. H., Li L. J. (2015). Nano Energy.

[cit4] Fatima J., Tahir M. B., Rehman A., Sagir M., Rafique M., Assiri M. A., Alzaid M. (2023). Mater. Sci. Eng., B.

[cit5] Landis M. E., Aufdembrink B. A., Chu P., Johnson I. D., Kirker G. W., Rubin M. K. (1991). J. Am. Chem. Soc..

[cit6] Khan K., Tareen A. K., Aslam M., Wang R., Zhang Y., Mahmood A., Guo Z. (2020). J. Mater. Chem. C.

[cit7] Raza A., Qumar U., Hassan J., Ikram M., Ul-Hamid A., Haider J., Ali S. (2020). Appl. Nanosci..

[cit8] Chaves A., Azadani J. G., Alsalman H., Da Costa D. R., Frisenda R., Chaves A. J., Low T. (2020). npj 2D Mater. Appl..

[cit9] Cao L., Zhou G., Wu Q., Yang S. A., Yang H. Y., Ang Y. S., Ang L. K. (2020). Phys. Rev. Appl..

[cit10] Xu W., Ke Y., Wang Z., Zhang W., Wee A. T. S. (2021). Surf. Sci. Rep..

[cit11] Zhang Y., Xu M., Zeng Q., Hao J., Li Y. (2023). Mater. Today Electronics.

[cit12] Butler S. Z., Hollen S. M., Cao L., Cui Y., Gupta J. A., Gutiérrez H. R., Goldberger J. E. (2013). ACS Nano.

[cit13] Fan F. R., Wang R., Zhang H., Wu W. (2021). Chem. Soc. Rev..

[cit14] Mehdi Aghaei S., Aasi A., Panchapakesan B. (2021). ACS Omega.

[cit15] Anasori B., Lukatskaya M. R., Gogotsi Y. (2017). Nat. Rev. Mater..

[cit16] Gupta A., Sakthivel T., Seal S. (2015). Prog. Mater. Sci..

[cit17] Wang G., Yang P., Batista E. R. (2020). Phys. Rev. Mater..

[cit18] Izyumskaya N., Demchenko D. O., Avrutin V., Özgür Ü., Morkoç H. (2014). Turk. J. Phys..

[cit19] Kang S., Lee D., Kim J., Capasso A., Kang H. S., Park J. W., Lee G. H. (2020). 2D Mater..

[cit20] Feng Y. P., Shen L., Yang M., Wang A., Zeng M., Wu Q., Chang C. R. (2017). Wiley Interdiscip. Rev.:Comput. Mol. Sci..

[cit21] Zhao M., Hao Y., Zhang C., Zhai R., Liu B., Liu W., Li H. (2022). Crystals.

[cit22] Lin Y. C., Torsi R., Younas R., Hinkle C. L., Rigosi A. F., Hill H. M., Robinson J. A. (2023). ACS Nano.

[cit23] Mikhailova M. P., Moiseev K. D., Yakovlev Y. P. (2019). Semiconductors.

[cit24] Joyce H. J., Gao Q., Tan H. H., Jagadish C., Kim Y., Zou J., Johnston M. B. (2011). Prog. Quantum Electron..

[cit25] Singh A. K., Zhuang H. L., Hennig R. G. (2014). Phys. Rev. B:Condens. Matter Mater. Phys..

[cit26] Buscema M., Island J. O., Groenendijk D. J., Blanter S. I., Steele G. A., van der Zant H. S., Castellanos-Gomez A. (2015). Chem. Soc. Rev..

[cit27] Zeng M., Xiao Y., Liu J., Yang K., Fu L. (2018). Chem. Rev..

[cit28] Lan C., Shi Z., Cao R., Li C., Zhang H. (2020). Nanoscale.

[cit29] Yu S., Wu X., Wang Y., Guo X., Tong L. (2017). Adv. Mater..

[cit30] Chakraborty S. K., Kundu B., Nayak B., Dash S. P., Sahoo P. K. (2022). iScience.

[cit31] Lo C.-L., Helfrecht B. A., He Y., Guzman D. M., Onofrio N., Zhang S., Weinstein D., Strachan A., Chen Z. (2020). J. Appl. Phys..

[cit32] Watson A. J., Lu W., Guimarães M. H., Stöhr M. (2021). 2D Mater..

[cit33] Wang X., Sun Y., Liu K. (2019). 2D Mater..

[cit34] Qi J., Wu Z., Wang W., Bao K., Wang L., Wu J., He Q. (2023). Int. J. Extreme Manuf..

[cit35] Li Z. H., Han J. N., Cao S. G., Zhang Z. H. (2023). Appl. Surf. Sci..

[cit36] Mohanta M. K., Kishore A., De Sarkar A. (2020). Nanotechnology.

[cit37] Albar A., Aravindh S. A. (2021). J. Phys.: Condens. Matter.

[cit38] Li Q., Meng J., Li Z. (2022). J. Mater. Chem. A.

[cit39] Xiong Y., Xu D., Feng Y., Zhang G., Lin P., Chen X. (2023). Adv. Mater..

[cit40] Su T., Li Y., Wang Q., Zhao W., Cao L., Ang Y. S. (2023). J. Phys. D: Appl. Phys..

[cit41] Nalwa H. S. (2020). RSC Adv..

[cit42] Li S., Wang F., Wang Y., Yang J., Wang X., Zhan X., Wang Z. (2024). Adv. Mater..

[cit43] Yang R., Fan J., Sun M. (2022). Front. Phys..

[cit44] Kim C., Moon I., Lee D., Choi M. S., Ahmed F., Nam S., Cho Y., Shin H.-J., Park S., Yoo W. J. (2017). ACS Nano.

[cit45] Guo Y., Liu D., Robertson J. (2015). ACS Appl. Mater. Interfaces.

[cit46] Kang J., Liu W., Sarkar D., Jena D., Banerjee K. (2014). Phys. Rev. X.

[cit47] Gong C., Colombo L., Wallace R. M., Cho K. (2014). Nano Lett..

[cit48] Khan H., Ashraf M. U., Idrees M., Din H. U., Nguyen C. V., Amin B. (2022). RSC Adv..

[cit49] Ashraf M. U., Khan H., Munawar M., Ud Din H., Idrees M., Bilal M., Saeed Y., Shafiq M., Amin B. (2022). Mater. Sci. Semicond. Process..

[cit50] Khan U., Ali B., Ullah H., Idrees M., Nguyen C., Amin B. (2024). Micro Nanostruct..

[cit51] Giannozzi P., Baroni S., Bonini N., Calandra M., Car R., Cavazzoni C., Ceresoli D., Chiarotti G. L., Cococcioni M., Dabo I., Dal Corso A., de Gironcoli S., Fabris S., Fratesi G., Gebauer R., Gerstmann U., Gougoussis C., Kokalj A., Lazzeri M., Martin-Samos L., Marzari N., Mauri F., Mazzarello R., Paolini S., Pasquarello A., Paulatto L., Sbraccia C., Scandolo S., Sclauzero G., Seitsonen A. P., Smogunov A., Umari P., Wentzcovitch R. M. (2009). J. Phys.: Condens. Matter.

[cit52] Perdew J. P., Burke K., Ernzerhof M. (1996). Phys. Rev. Lett..

[cit53] Wang V., Xu N., Liu J. C., Tang G., Geng W. T. (2021). Comput. Phys. Commun..

[cit54] Gaillac R., Pullumbi P., Coudert F.-X. (2016). J. Phys.: Condens. Matter.

[cit55] Yuan R., Napoli J. A., Yan C., Marsalek O., Markland T. E., Fayer M. D. (2019). ACS Cent. Sci..

[cit56] Malyi O. I., Sopiha K. V., Persson C. (2019). ACS Appl. Mater. Interfaces.

[cit57] Kresse G., Furthmüller J. (1996). Phys. Rev. B:Condens. Matter Mater. Phys..

[cit58] Togo A., Oba F., Tanaka I. (2008). Phys. Rev. B:Condens. Matter Mater. Phys..

[cit59] Bardeen J. (1947). Phys. Rev..

[cit60] Liu Y., Stradins P., Wei S.-H. (2016). Sci. Adv..

[cit61] Liu B., Wu L., Zhao Y. Q., Wang L. Z., Caii M. Q. (2016). Phys.
Chem. Chem. Phys..

[cit62] Guo N., Fan X., Chen Z., Luo Z., Hu Y., An Y., Ma S. (2020). Comput. Mater. Sci..

[cit63] Xie M., Zhang S., Cai B., Zhu Z., Zou Y., Zeng H. (2016). Nanoscale.

[cit64] Zhang Y., Tong Z., Pecchia A., Yam C., Dumitric T., Frauenheim T. (2022). Nanoscale.

[cit65] Ding Y., Wang Y., Ni J., Shi L., Shi S., Tang W. (2011). Phys. B.

[cit66] Onat B., Hallioglu L., Ipek S., Durgun E. (2017). J. Phys. Chem. C.

[cit67] Kolavada H., Trivedi D. J., Gajjar P. N., Gupta S. K. (2023). J. Energy Storage.

[cit68] Amin B., Singh N., Schwingenschlögl U. (2015). Phys. Rev. B:Condens. Matter Mater. Phys..

[cit69] Barik G., Pal S. (2020). Phys. Chem. Chem. Phys..

[cit70] Zhu X., Jiang H., Zhang Y., Wang D., Fan L., Chen Y., Qu X., Yang L., Liu Y. (2023). Molecules.

[cit71] Ashraf M. U., Xu Y., Yar M., Ni X., Tian F. (2024). Mater. Sci. Semicond. Process..

[cit72] Lei C., Ma Y., Xu X., Zhang T., Huang B., Dai Y. (2019). J. Phys. Chem. C.

[cit73] Hou Z., Xiao Y., Zhao L. D. (2020). Nanoscale.

[cit74] Rezaee S., Boochani A., Majidiyan M., Ghaderi A., Solaymani S., Naseri M. (2014). Rare Met..

[cit75] Zhang X., Liu B., Gao L., Yu H., Liu X., Du J., Zhang Y. (2021). Nat. Commun..

[cit76] Chen R. S., Ding G., Zhou Y., Han S. T. (2021). J. Mater. Chem.C.

[cit77] Zhang Z., Gong Y., Zou X., Liu P., Yang P., Shi J., Zhang Y. (2018). ACS Nano.

[cit78] Ai W., Shi Y., Hu X., Yang J., Sun L. (2023). ACS Appl. Electron. Mater..

[cit79] Liao J. F., Wu W. Q., Jiang Y., Zhong J. X., Wang L., Kuang D. B. (2020). Chem. Soc. Rev..

[cit80] Khan A., Din H. U., Idrees M., Khan F., Alrebdi T. A., Nguyen C. V., Shafiq M., Amin B. (2019). Phys. Lett. A.

[cit81] Ahmadi M., Collins L., Higgins K., Kim D., Lukosi E., Kalinin S. V. (2019). ACS Appl. Mater. Interfaces.

[cit82] Liu C., Xu Y., Noh Y. Y. (2015). Mater. Today.

[cit83] Nguyen H. T., Obeid M. M., Bafekry A., Idrees M., Vu T. V., Phuc H. V., Nguyen C. V. (2020). Phys. Rev. B.

[cit84] Pham K. D., Hieu N. N., Phuc H. V., Fedorov I. A., Duque C. A., Amin B., Nguyen C. V. (2018). Appl. Phys. Lett..

[cit85] Kiguchi M., Nakayama M., Fujiwara K., Ueno K., Shimada T., Saiki K. (2003). Jpn. J. Appl. Phys..

[cit86] Yue X., Fan J., Xiang Q. (2022). Adv. Funct. Mater..

[cit87] Ben Aziza Z., Henck H., Pierucci D., Silly M. G., Lhuillier E., Patriarche G., Sirotti F., Eddrief M., Ouerghi A. (2016). ACS Nano.

[cit88] Pierucci D., Henck H., Avila J., Balan A., Naylor C. H., Patriarche G., Dappe Y. J., Silly M. G., Sirotti F., Johnson A. C., Asensio M. C., Ouerghi A. (2016). Nano Lett..

